# Revolution in malaria detection: unveiling current breakthroughs and tomorrow’s possibilities in biomarker innovation

**DOI:** 10.1097/MS9.0000000000002383

**Published:** 2024-07-17

**Authors:** Emmanuel Ifeanyi Obeagu, G. I.A. Okoroiwu, N. I. Ubosi, Getrude U. Obeagu, Hope Onohuean, Tukur Muhammad, Teddy C. Adias

**Affiliations:** a Department of Medical Laboratory Science, Kampala International University; b Department of Science Education & Educational Foundations, Faculty of Education Kampala International University Western Campus; c School of Nursing Science, Kampala International University; d Biopharmaceutics Unit, Department of Pharmacology and Toxicology, School of Pharmacy, Kampala International University, Kampala; e Biomolecules, Metagenomics, Endocrine and Tropical Disease Research Group (BMETDREG), Kampala International University, Western Campus, Ishaka-Bushenyi, Uganda; f Department of Public Health Science, Faculty of Health Sciences, National Open University of Nigeria, Jabi, Abuja; g Department of Haematology and Blood Transfusion Science, Faculty of Medical Laboratory Science, Federal University Otuoke, Bayelsa State, Nigeria

**Keywords:** biomarkers, diagnosis, malaria, monitoring, treatment

## Abstract

The ongoing battle against malaria has seen significant advancements in diagnostic methodologies, particularly through the discovery and application of novel biomarkers. Traditional diagnostic techniques, such as microscopy and rapid diagnostic tests, have their limitations in terms of sensitivity, specificity, and the ability to detect low-level infections. Recent breakthroughs in biomarker research promise to overcome these challenges, providing more accurate, rapid, and non-invasive detection methods. These advancements are critical in enhancing early detection, guiding effective treatment, and ultimately reducing the global malaria burden. Innovative approaches in biomarker detection are leveraging cutting-edge technologies like next-generation sequencing, proteomics, and metabolomics. These techniques have led to the identification of new biomarkers that can be detected in blood, saliva, or urine, offering less invasive and more scalable options for widespread screening. For instance, the discovery of specific volatile organic compounds in the breath of infected individuals presents a revolutionary non-invasive diagnostic tool. Additionally, the integration of machine learning algorithms with biomarker data is enhancing the precision and predictive power of malaria diagnostics, making it possible to distinguish between different stages of infection and identify drug-resistant strains. Looking ahead, the future of malaria detection lies in the continued exploration of multi-biomarker panels and the development of portable, point-of-care diagnostic devices. The incorporation of smartphone-based technologies and wearable biosensors promises to bring real-time monitoring and remote diagnostics to even the most resource-limited settings.

## Introduction

Highlights

- *Novel biomarkers*: Highlight recent advancements in identifying novel biomarkers for malaria detection, such as host-derived factors, parasite antigens, and metabolic signatures, offering more sensitive and specific diagnostic tools.
- *Point-of-care testing*: Discuss the emergence of rapid diagnostic tests (RDTs) and portable molecular diagnostic devices that enable prompt and accurate malaria diagnosis at the point of care, particularly in resource-limited settings.
- *Next-generation technologies*: Explore innovative technologies like loop-mediated isothermal amplification (LAMP), polymerase chain reaction (PCR), and digital microfluidics, which revolutionize malaria detection by enhancing sensitivity, specificity, and scalability.
- *Multi-modal approaches*: Highlight the potential of combining multiple biomarkers or diagnostic techniques, such as serological assays, nucleic acid amplification, and imaging modalities, to improve the accuracy and reliability of malaria diagnosis across different transmission settings.
- *Biosensor platforms*: Showcase the development of biosensor platforms based on nanotechnology, microfluidics, and miniaturized electronic systems, enabling real-time, label-free detection of malaria parasites or biomarkers in biological samples.
- *Big data and machine learning*: Discuss the integration of big data analytics and machine learning algorithms to identify patterns and correlations in large datasets of clinical, epidemiological, and genomic information, facilitating early malaria detection and surveillance.
- *Targeted therapeutics*: Explore how biomarker discovery drives the development of targeted antimalarial therapies, such as drugs that selectively inhibit parasite-specific metabolic pathways or exploit host immune responses for parasite clearance.
- *Field-deployable solutions*: Emphasize the importance of developing field-deployable diagnostic tools that are robust, user-friendly, and cost-effective, enabling widespread adoption in endemic regions and remote areas with limited healthcare infrastructure.
- *Public health impact*: Evaluate the potential public health impact of biomarker-driven innovations in malaria detection, including reduced morbidity and mortality, enhanced surveillance capabilities, and informed decision-making for malaria control and elimination strategies.

Malaria remains one of the most devastating infectious diseases globally, causing significant morbidity and mortality, particularly in tropical and subtropical regions. The disease, caused by *Plasmodium* parasites and transmitted through the bites of infected *Anopheles* mosquitoes, has challenged public health systems for centuries. Despite substantial efforts to control and eliminate malaria, it continues to pose a major health threat, with hundreds of millions of cases and hundreds of thousands of deaths reported annually. The need for more effective and efficient diagnostic methods has never been more critical, as early and accurate detection is pivotal in managing and reducing the disease’s impact^1^. Traditional malaria diagnostic methods, such as microscopy and rapid diagnostic tests (RDTs), have played crucial roles in malaria control programs. Microscopy, considered the gold standard for malaria diagnosis, involves the examination of stained blood smears under a microscope to identify the presence of *Plasmodium* parasites. While highly specific, this method requires skilled personnel, significant time, and laboratory infrastructure, limiting its applicability in many resource-limited settings. RDTs, on the other hand, provide a quicker alternative by detecting specific antigens derived from *Plasmodium* species in a patient’s blood. However, RDTs vary in sensitivity and specificity, and their performance can be affected by factors such as parasite density and genetic variations in *Plasmodium* strains^2^. In recent years, the scientific community has made remarkable strides in identifying and utilizing novel biomarkers for malaria detection. Biomarkers, defined as biological molecules that indicate a particular disease state, offer the potential for more sensitive, specific, and rapid diagnostic methods. The advent of technologies such as next-generation sequencing (NGS), proteomics, and metabolomics has enabled the discovery of a plethora of new biomarkers associated with malaria infection. These biomarkers, detectable in various biological samples such as blood, saliva, urine, and even breath, represent a significant leap forward in diagnostic capabilities^3^.

One of the groundbreaking developments in this field is the identification of volatile organic compounds (VOCs) in the breath of malaria-infected individuals. VOCs are metabolic byproducts that can be detected non-invasively using advanced analytical techniques like gas chromatography–mass spectrometry (GC–MS). The ability to diagnose malaria through breath analysis not only simplifies the testing process but also enhances patient comfort and compliance, which is particularly important in pediatric populations. This innovation underscores the shift toward less invasive diagnostic methods that are easier to deploy in field settings^4^. Proteomic and metabolomic studies have also uncovered numerous protein and metabolite biomarkers that are differentially expressed during malaria infection. For example, specific proteins released by *Plasmodium* parasites into the host’s bloodstream can serve as reliable indicators of infection. Similarly, alterations in the host’s metabolic profile in response to the parasite can be detected using metabolomic approaches, providing a comprehensive picture of the disease state. These insights have paved the way for the development of highly sensitive diagnostic assays capable of detecting malaria at very low parasite densities^5^. The integration of machine learning (ML) and artificial intelligence (AI) with biomarker data is another exciting frontier in malaria diagnostics. ML algorithms can analyze complex datasets to identify patterns and correlations that may not be apparent through traditional analytical methods. By training these algorithms on extensive biomarker profiles, researchers can develop predictive models that enhance diagnostic accuracy and reliability. Such models can also assist in distinguishing between different stages of malaria infection and identifying cases of drug-resistant malaria, which is crucial for effective disease management and treatment planning^6^.

Looking forward, the future of malaria diagnostics lies in the continued advancement and integration of these innovative technologies. Multi-biomarker panels, which combine several biomarkers to improve diagnostic precision, are expected to play a significant role. These panels can provide a more holistic view of the infection, capturing various aspects of the host–parasite interaction. Furthermore, the development of portable, point-of-care (POC) diagnostic devices incorporating these biomarker panels holds promise for real-time, on-site malaria testing, particularly in remote and underserved areas^7^. The potential of smartphone-based technologies and wearable biosensors in malaria diagnostics is also being explored. Smartphones equipped with diagnostic apps and connected to portable devices can analyze biomarker data and provide immediate results. Wearable biosensors that continuously monitor physiological and biochemical parameters can detect early signs of malaria infection, facilitating timely intervention. These technologies not only enhance diagnostic capabilities but also support the broader goal of digital health and telemedicine, making healthcare more accessible and responsive^8^. The successful translation of these scientific innovations into practical diagnostic tools requires collaborative efforts across various sectors. Researchers, healthcare providers, technology developers, and policymakers must work together to address challenges related to scalability, cost, and implementation. Ensuring that these advanced diagnostic methods are affordable and accessible to all populations, particularly those in low-resource settings, is essential for their widespread adoption and impact^6^.

## Aim

The aim of ‘Revolution in malaria detection: unveiling current breakthroughs and tomorrow’s possibilities in biomarker innovation’ is to provide a comprehensive overview of the latest advancements in malaria diagnosis, with a focus on biomarker innovation. This review aims to:

1. Summarize the current landscape of malaria detection methods, including microscopy, rapid diagnostic tests (RDTs), molecular assays, and their limitations.
2. Explore the role of biomarkers in malaria diagnosis, highlighting commonly used biomarkers such as histidine-rich protein 2 (HRP2), lactate dehydrogenase (LDH), aldolase, and *Plasmodium* DNA/RNA.
3. Discuss emerging technologies and innovations in malaria detection, such as CRISPR-based diagnostics, nanotechnology, metabolomics, and artificial intelligence (AI).
4. Evaluate the challenges and opportunities in biomarker innovation for malaria detection, including sensitivity, cost, standardization, and infrastructure constraints.
5. Provide recommendations for future research directions, collaboration, and policy interventions to accelerate the translation of biomarker innovation into clinical practice and public health impact.

## Rationale

The rationale behind ‘Revolution in malaria detection: unveiling current breakthroughs and tomorrow’s possibilities in biomarker innovation’ stems from the urgent need to improve malaria diagnosis, a critical component of malaria control and elimination efforts worldwide. Despite significant progress in malaria prevention and treatment, accurate and timely diagnosis remains a challenge, particularly in resource-limited settings where the burden of malaria is highest. Traditional diagnostic methods, such as microscopy and RDTs, have limitations in terms of sensitivity, specificity, and scalability, hindering effective case management and surveillance. Biomarker innovation holds immense promise for revolutionizing malaria detection by offering sensitive, specific, and rapid diagnostic solutions that can complement or even replace existing methods. Biomarkers, such as histidine-rich protein 2 (HRP2), lactate dehydrogenase (LDH), aldolase, and *Plasmodium* DNA/RNA, provide valuable insights into parasite presence, species differentiation, disease severity, and treatment response. Moreover, emerging technologies, including CRISPR-based diagnostics, nanotechnology, metabolomics, and artificial intelligence (AI), offer unprecedented opportunities for enhancing diagnostic accuracy, accessibility, and affordability. The rationale for this review is to critically evaluate the current state of malaria diagnosis, highlight recent breakthroughs and innovations in biomarker discovery and technology development, and identify challenges and opportunities for translating biomarker innovation into clinical practice and public health impact. By synthesizing the latest evidence and insights from multidisciplinary fields, this review aims to inform researchers, policymakers, and healthcare stakeholders about the transformative potential of biomarker innovation in advancing malaria control and elimination goals. Ultimately, the goal is to catalyze collaborative efforts and strategic investments toward realizing a future where accurate and accessible malaria diagnosis is within reach for all.

## Review methodology

### Search strategy

1. *Database selection:* A systematic search was conducted in academic databases including PubMed, Scopus, Web of Science, and Google Scholar to identify relevant peer-reviewed articles, reviews, and conference proceedings published between January 2010 and December 2023.
2. *Search terms:* The search strategy utilized a combination of keywords related to malaria (“malaria”, “Plasmodium”, “malaria diagnosis”) and biomarker innovation (“biomarker”, “diagnostic biomarker”, “biomarker innovation”, “molecular diagnostics”).
3. *Inclusion criteria:* Studies included in the review met the following criteria: (a) focused on malaria diagnosis and biomarker innovation, (b) published in English, (c) peer-reviewed articles or reviews, (d) conducted in human populations or relevant animal models.
4. *Exclusion criteria:* Studies were excluded if they were not related to malaria diagnosis or biomarker innovation, not published in English, or not accessible in full-text format.

### Quality assessment

1. *Critical appraisal:* The quality of included studies was assessed based on study design, methodology, sample size, and reporting quality.
2. *Risk of bias:* Potential sources of bias, including selection bias, measurement bias, and confounding factors, were evaluated to ensure the validity and reliability of study findings.
3. *Strength of evidence:* The strength of evidence from included studies was evaluated based on the relevance, rigor, and consistency of study findings.

### Biomarkers for malaria diagnosis

Malaria is a life-threatening infectious disease caused by *Plasmodium* parasites, transmitted to humans through the bite of infected *Anopheles* mosquitoes^9^. Early and accurate diagnosis of malaria is essential for prompt treatment and the control of disease transmission^10^. While several diagnostic methods are available, biomarkers have become instrumental in enhancing the precision and efficiency of malaria diagnosis^11^. Biomarkers are specific molecules, proteins, or other measurable indicators that can be detected in a patient’s body fluids or tissues. In the context of malaria, biomarkers can reveal the presence of the parasite, the type of *Plasmodium* species responsible for the infection, and even the stage of the disease^12,13^. Microscopy, the traditional method for malaria diagnosis, involves examining blood smears under a microscope to detect the parasite. While it requires trained personnel, it is still widely used in resource-limited settings^14^. Rapid Diagnostic Tests (RDTs) are immunochromatographic tests that detect specific malaria antigens in a patient’s blood. They are easy to use, provide quick results, and are particularly suitable for remote or low-resource areas^15^. Polymerase Chain Reaction (PCR): PCR-based assays are highly sensitive and can detect even low levels of parasites in a patient’s blood. They can also differentiate between *Plasmodium* species and strains, aiding in the selection of appropriate treatment^16^. Loop-Mediated Isothermal Amplification (LAMP) is a robust molecular method for detecting *Plasmodium* DNA that does not require sophisticated equipment, making it suitable for field applications^17^. Antibodies and Antigens: Detection of specific antibodies or antigens in a patient’s blood can be indicative of a current or past infection. This approach can be especially useful in detecting asymptomatic infections and estimating disease prevalence^18^. The measurement of cytokines, such as interferon-gamma and interleukins, can provide information about the immune response to malaria, aiding in diagnosis and monitoring^19^. Breath and Sweat Biomarkers: Emerging research suggests that volatile organic compounds in breath or sweat can serve as non-invasive indicators of malaria infection. This approach shows promise for future diagnostic tools^20^. Optical and Acoustic Sensors: Innovative technologies utilizing optical and acoustic properties of blood samples are being explored for malaria diagnosis, offering potential for rapid and non-invasive testing^21^. The choice of diagnostic method depends on factors such as the region’s endemicity, the resources available, and the specific goals of the diagnosis (e.g. screening, confirmation, or research)^22^. Additionally, accurate diagnosis is crucial in identifying drug-resistant strains and assessing the effectiveness of treatment. Biomarkers have revolutionized malaria diagnosis, offering a range of methods that are increasingly sensitive, specific, and accessible. These tools are critical not only for individual patient care but also for epidemiological surveillance, enabling health authorities to track and respond to malaria outbreaks effectively^23,24^. As research continues to advance, the future of malaria diagnosis promises even more innovative and accurate biomarker-based approaches, ultimately contributing to the global efforts to control and eliminate this devastating disease.

### Biomarkers for malaria severity

Malaria, caused by *Plasmodium* parasites, can manifest in various forms, from mild and uncomplicated cases to severe and life-threatening infections. Biomarkers, specific measurable indicators in the body, play a crucial role in assessing the severity of malaria^5,25^. Understanding the severity of the disease is essential for guiding treatment decisions, as well as for monitoring the patient’s response to therapy. Hemoglobin levels are a fundamental biomarker used to gauge the severity of malaria. Malaria parasites invade red blood cells, leading to their destruction and causing anemia^26,27^. Severe malaria often results in profound anemia, where hemoglobin levels drop significantly, potentially requiring blood transfusions to manage the condition^28^. Thrombocytopenia, a low platelet count, is a common finding in severe malaria cases. Platelets play a crucial role in blood clotting, and a decrease in their number can lead to bleeding complications^29^. Monitoring platelet counts is vital for assessing the risk of hemorrhagic events in severe malaria^30^. In severe malaria, the immune response becomes dysregulated, leading to the release of pro-inflammatory cytokines, such as tumor necrosis factor (TNF) and interleukin-6 (IL-6)^31^. Elevated levels of these inflammatory markers are associated with the severity of the disease and may contribute to complications like cerebral malaria^32^. Metabolomics and proteomics are emerging fields that focus on studying the metabolic and protein profile of individuals with malaria^33^. Changes in metabolites and proteins in response to the infection can provide insights into the severity and progression of the disease, potentially identifying novel biomarkers^34^. Endothelial cells lining blood vessels are affected in severe malaria. Biomarkers related to endothelial dysfunction, such as von Willebrand factor (vWF) and angiopoietin-2, can indicate vascular damage^35^. Monitoring endothelial biomarkers can help assess the risk of complications associated with microvascular occlusion^36^. Abnormalities in the coagulation system are frequently observed in severe malaria^37^. Biomarkers like D-dimer and fibrinogen can reflect coagulation disturbances. Coagulation biomarkers are important for identifying patients at risk of coagulopathy and thromboembolic events^38^. The assessment of malaria severity is a dynamic process that requires a combination of clinical evaluation and biomarker measurements. These biomarkers not only help in determining the level of disease severity but also in tailoring treatment strategies, monitoring the patient’s response to therapy, and predicting potential complications^39^. While these biomarkers have greatly improved our understanding of malaria severity, ongoing research aims to identify additional markers and develop more accurate, sensitive, and specific indicators. Ultimately, a comprehensive understanding of malaria severity biomarkers is instrumental in reducing mortality and improving the management of severe malaria cases, particularly in regions with high disease burden.

### Biomarkers for monitoring treatment efficacy of malaria

Monitoring the efficacy of malaria treatment is essential to ensure that patients receive the most appropriate and effective therapy, as well as to detect drug resistance early. Biomarkers, specific indicators or measurable molecules, play a vital role in assessing the response of malaria infections to treatment^40,41^. Monitoring the rate at which malaria parasites are cleared from the patient’s bloodstream is a fundamental method for assessing treatment efficacy^42^. Biomarkers such as parasite density, measured through microscopy or molecular methods, provide insight into the effectiveness of antimalarial drugs^43^. A rapid reduction in parasite density is indicative of successful treatment. With the emergence of drug-resistant *Plasmodium* strains, it is crucial to identify markers associated with resistance to specific antimalarial drugs^44^. Genetic biomarkers, such as specific mutations in the *Plasmodium* genome, can reveal resistance to drugs like chloroquine or sulfadoxine-pyrimethamine. Monitoring these genetic changes helps guide treatment choices^45^. Proteomic and metabolomic biomarkers provide a comprehensive view of the molecular changes occurring in response to treatment. Changes in the levels of specific proteins or metabolites, such as hemozoin or amino acids, can indicate the effect of treatment on the parasite’s metabolic pathways and help evaluate drug efficacy^46^. Immunological biomarkers, such as antibody responses and T-cell reactions, reflect the patient’s immune system’s interaction with the malaria parasite^47^. Monitoring the development of specific antibodies against *Plasmodium* antigens and assessing T-cell responses can provide insights into the patient’s immune response and the effectiveness of treatment^48^. Inflammatory markers like C-reactive protein (CRP) and pro-inflammatory cytokines (e.g. IL-6 and TNF) can be indicative of the patient’s systemic response to malaria and treatment^49^. Monitoring changes in these markers helps assess the resolution of inflammation and the overall recovery of the patient. For certain *Plasmodium* species, such as *P. vivax* and *P. ovale*, the potential for relapse exists even after successful treatment of the acute infection^50^. Monitoring specific genetic markers or PCR assays can predict the risk of relapse, ensuring appropriate follow-up and radical cure^51^. Clinical biomarkers such as fever resolution, resolution of symptoms, and hemoglobin recovery are practical indicators for treatment efficacy^52^. These clinical parameters are readily accessible and provide valuable information for healthcare providers^52^. Monitoring the treatment efficacy of malaria is critical for ensuring that patients receive the most effective and timely care, while also contributing to the global effort to combat drug-resistant strains^53^. Biomarkers serve as valuable tools in this endeavor, offering insights into the response of the parasite, the patient’s immune system, and the overall progress toward recovery^54^. As research advances, the development of more precise and sensitive biomarkers continues to enhance our ability to monitor and manage malaria treatment effectively.

### Biomarkers for diagnosis of malaria

The diagnosis of malaria often relies on the detection of specific biomarkers associated with the presence of the malaria parasite^55^. Various biomarkers from both the host and the parasite are utilized in different diagnostic methods. Here are some key biomarkers commonly used for the diagnosis of malaria:

#### Histidine-rich protein 2 (HRP2)

Histidine-rich protein 2 (HRP2) is a protein produced by the *Plasmodium falciparum* parasite, which is the deadliest of the malaria-causing species. HRP2 is released into the bloodstream of infected individuals and has become a crucial target for malaria diagnostics due to its abundance and the ease with which it can be detected. HRP2 is the primary target for many RDTs for malaria. These tests typically involve a lateral flow assay, where a blood sample is applied to a test strip. If HRP2 is present in the sample, it binds to specific antibodies on the test strip, producing a visible line indicating a positive result. RDTs targeting HRP2 are widely used because they are quick, easy to perform, and do not require specialized equipment or training, making them ideal for field conditions and resource-limited settings. HRP2-based RDTs are highly sensitive, capable of detecting low levels of parasitemia, which is critical for early diagnosis and treatment. These tests are simple and can be administered by minimally trained personnel, which is vital in rural and remote areas. Results are typically available within 15–30 min, facilitating rapid decision-making for treatment. One of the significant challenges with HRP2-based diagnostics is the emergence of *P. falciparum* strains with deletions in the hrp2 gene. These deletions render the RDTs ineffective as the tests fail to detect the presence of the parasite, leading to false negatives. This issue is particularly concerning in areas with high prevalence of hrp2 deletions. HRP2 can persist in the bloodstream for weeks after the infection has been cleared, which can lead to false-positive results. This limitation affects the ability of HRP2-based RDTs to accurately reflect current infection status. The sensitivity and specificity of HRP2-based RDTs can vary depending on the brand and manufacturing quality. Ensuring consistent performance across different RDTs is a continuing challenge^19^.

Plasmodium lactate dehydrogenase (pLDH) is another protein produced by malaria parasites and is used in some RDTs. pLDH-based tests can detect multiple *Plasmodium* species and are not affected by hrp2 deletions, making them a useful alternative. Some RDTs combine HRP2 and pLDH detection to improve diagnostic accuracy and reduce the likelihood of false negatives associated with hrp2 deletions. Techniques like PCR and LAMP offer higher sensitivity and can detect hrp2 deletions, but they require more resources and technical expertise. Monitoring the prevalence of hrp2 deletions through genomic surveillance helps in understanding the spread and impact of these deletions on diagnostic accuracy. This information is crucial for adapting diagnostic strategies in affected regions. Identifying and validating new biomarkers for malaria that are not subject to genetic deletions or variability can provide more reliable diagnostic targets. Efforts to standardize and improve the manufacturing quality of RDTs can help ensure consistent performance. Research into more robust and durable test formats is also ongoing^20^.

#### Lactate dehydrogenase (LDH)

Lactate dehydrogenase (LDH) is an enzyme involved in the glycolytic pathway, facilitating the conversion of pyruvate to lactate. In the context of malaria, LDH is produced by *Plasmodium* parasites during their asexual reproduction in red blood cells. Since LDH is released into the host’s bloodstream during infection, it serves as a useful biomarker for malaria diagnosis. Unlike HRP2, which is specific to *P. falciparum*, LDH is present in all *Plasmodium* species, making it a versatile target for diagnostic tests. LDH is targeted in some RDTs designed to diagnose malaria. These tests work similarly to HRP2-based RDTs, utilizing lateral flow immunoassays to detect the presence of *Plasmodium* LDH (pLDH) in a patient’s blood sample. The binding of pLDH to specific antibodies on the test strip results in a visible line, indicating a positive result. pLDH-based RDTs can detect all *Plasmodium* species, including *P. falciparum*, *P. vivax*, *P. malariae*, and *P. ovale*. This broad detection range is particularly useful in regions where multiple *Plasmodium* species are endemic. Unlike HRP2, which can be absent in some *P. falciparum* strains due to genetic deletions, pLDH is consistently present in all species. This reduces the risk of false negatives due to genetic variability. pLDH levels correlate with the presence of viable parasites, making it a reliable indicator of active infection. Once the infection is cleared, pLDH levels drop quickly, which minimizes the likelihood of false positives from previous infections^21^.

While pLDH-based RDTs are generally effective, their sensitivity can be lower compared to HRP2-based tests, particularly at low parasite densities. This can impact their ability to detect early or asymptomatic infections. RDTs, including those based on pLDH, can be affected by extreme temperatures, which may degrade the antibodies used in the test and reduce accuracy. Proper storage and handling are essential to maintain test performance. Although less resource-intensive than molecular methods, pLDH-based RDTs still require blood samples and appropriate handling, which can be challenging in some remote or resource-poor settings. Combining pLDH with HRP2 in a single test can enhance diagnostic accuracy by leveraging the strengths of both biomarkers. This approach mitigates the risk of false negatives due to hrp2 deletions and improves overall sensitivity. Techniques such as PCR and LAMP offer higher sensitivity and specificity. These methods can detect low levels of parasitemia and differentiate between *Plasmodium* species. While more resource-intensive, they provide valuable confirmation of RDT results. Advances in proteomics and metabolomics are identifying new biomarkers for malaria that can complement or replace pLDH. These biomarkers can provide additional diagnostic targets and improve the robustness of diagnostic assays. Efforts are underway to develop more sensitive pLDH-based tests that can detect lower levels of parasitemia, improving early detection and diagnosis of asymptomatic cases. Developing more stable formulations of pLDH-based RDTs that can withstand temperature variations and have a longer shelf life is critical for their deployment in diverse field conditions. Integrating pLDH-based diagnostics with digital health technologies, such as smartphone apps and portable readers, can facilitate data collection, real-time analysis, and remote monitoring. This integration can improve diagnostic accuracy and enable better tracking of malaria cases^22^.

#### Aldolase

Aldolase is an enzyme involved in the glycolytic pathway, catalyzing the conversion of fructose-1,6-bisphosphate into glyceraldehyde-3-phosphate and dihydroxyacetone phosphate. In the context of malaria, *Plasmodium* aldolase is an important enzyme produced by all species of the malaria-causing parasite. Given its presence in all *Plasmodium* species, aldolase serves as a potential biomarker for malaria diagnosis. Aldolase is targeted in some RDTs designed to detect malaria. These tests typically involve a lateral flow immunoassay format, where a blood sample is applied to a test strip. If *Plasmodium* aldolase is present, it binds to specific antibodies on the test strip, producing a visible line that indicates a positive result. Aldolase-based RDTs are often used in combination with other antigen targets, such as HRP2, to improve diagnostic accuracy. Aldolase is produced by all *Plasmodium* species, making aldolase-based RDTs capable of detecting *P. falciparum*, *P. vivax*, *P. malariae*, and *P. ovale*. This broad detection range is particularly beneficial in regions where multiple *Plasmodium* species coexist. Similar to pLDH, aldolase levels correlate with the presence of viable parasites, providing a reliable indicator of active infection. This helps distinguish between current and past infections. Combining aldolase detection with HRP2 in a single test improves overall diagnostic performance by leveraging the strengths of both biomarkers and mitigating the issue of HRP2 deletions in some *P. falciparum* strains^23^.

Aldolase-based RDTs may have lower sensitivity compared to HRP2-based tests, particularly at low parasite densities. This can affect their ability to detect early or asymptomatic infections. There is potential for cross-reactivity with human aldolase or aldolase from other pathogens, which can lead to false-positive results. Ensuring specific binding to *Plasmodium* aldolase is critical. Like other RDTs, aldolase-based tests can be affected by extreme temperatures, which may degrade the antibodies used in the test and reduce accuracy. Proper storage and handling are essential to maintain test performance. Combining aldolase with other biomarkers, such as HRP2 and pLDH, in a single test can enhance diagnostic accuracy and sensitivity. This multi-target approach provides a more comprehensive diagnostic solution. Techniques like PCR and LAMP offer higher sensitivity and specificity. These methods can detect low levels of parasitemia and provide species differentiation. While more resource-intensive, they serve as valuable confirmatory tests. Advances in proteomics and metabolomics are identifying new biomarkers for malaria that can complement or replace aldolase. These biomarkers can offer additional diagnostic targets and improve the robustness of diagnostic assays^24^.

#### Plasmodium DNA/RNA

Plasmodium DNA and RNA serve as fundamental targets for molecular diagnostics of malaria. These nucleic acids carry the genetic information of the *Plasmodium* parasites, providing a direct and specific means of detecting the presence of the parasite in infected individuals. *PCR (Polymerase Chain Reaction)* amplifies specific regions of *Plasmodium* DNA, enabling the detection and identification of the parasite. Various PCR-based assays, including conventional PCR, nested PCR, and quantitative real-time PCR (qPCR), are used for malaria diagnosis. These assays can differentiate between *Plasmodium* species and quantify parasite load in clinical samples. *LAMP (Loop-Mediated Isothermal Amplification)* is an isothermal amplification technique that amplifies DNA under constant temperature conditions. It offers rapid and sensitive detection of *Plasmodium* DNA and is suitable for field-based applications due to its simplicity and minimal equipment requirements. While less common than DNA-based methods, RNA-based assays can also be used for malaria diagnosis. Reverse transcription PCR (RT-PCR) amplifies RNA transcripts, allowing the detection of active transcriptional activity in the parasite. RNA-based assays can provide insights into parasite viability and gene expression profiles^25^.

Molecular methods targeting *Plasmodium* DNA/RNA offer high sensitivity, enabling the detection of low parasite densities and asymptomatic infections. These assays are highly specific to *Plasmodium* species, minimizing cross-reactivity with other pathogens and reducing false-positive results. PCR-based assays can differentiate between different *Plasmodium* species, such as *P. falciparum*, *P. vivax*, *P. malariae*, *P. ovale*, and *P. knowlesi*, allowing for accurate species identification. PCR-based methods require specialized laboratory equipment and trained personnel, limiting their accessibility in resource-limited settings. The cost of molecular diagnostics can be higher compared to RDTs, posing a barrier to widespread implementation in endemic regions. DNA/RNA extraction from clinical samples can be challenging, especially in settings with limited access to laboratory facilities and quality control measures. Development of *Point-of-Care (POC)* molecular diagnostic platforms that are portable, user-friendly, and affordable could facilitate decentralized testing and improve access to molecular diagnostics in remote areas. Multiplex PCR assays capable of detecting multiple *Plasmodium* species and drug resistance markers in a single reaction could streamline diagnostic workflows and improve efficiency. Integration of molecular diagnostic data with surveillance systems and digital health platforms can enhance real-time monitoring of malaria epidemiology, aiding in disease surveillance and control efforts^5^.

### Circulating parasite biomarkers

Microscopy remains a traditional method for directly observing the presence of the malaria parasite in blood smears. Giemsa-stained blood films are examined under a microscope to identify and quantify parasites.

### Cytokines and chemokines

Cytokines and chemokines are key signaling molecules involved in the immune response against malaria infection. They play critical roles in orchestrating both protective immunity and immunopathology, influencing the outcome of infection. Understanding the dynamic interplay between cytokines, chemokines, and immune cells is essential for elucidating malaria pathogenesis and developing effective therapeutic interventions. Cytokines such as tumor necrosis factor-alpha (TNF-α), interleukin-1β (IL-1β), and interleukin-6 (IL-6) are produced in response to malaria infection, contributing to the activation of innate immune cells and the induction of fever and inflammation. While these cytokines are essential for controlling parasitemia, dysregulated production can lead to immunopathology and severe malaria syndromes. Anti-inflammatory cytokines, including interleukin-10 (IL-10) and transforming growth factor-beta (TGF-β), act to dampen the pro-inflammatory response and limit tissue damage. They play crucial roles in modulating immune activation and preventing excessive inflammation and immunopathology. Chemokines are chemotactic cytokines that attract immune cells to sites of infection and inflammation. Chemokines such as CCL2 (monocyte chemoattractant protein-1) and CXCL8 (interleukin-8) recruit monocytes, neutrophils, and other leukocytes to the site of malaria infection, where they contribute to parasite clearance and tissue damage^26^.

Dysregulation of cytokine and chemokine responses is implicated in the pathogenesis of cerebral malaria, a severe complication of *P. falciparum* infection. Elevated levels of pro-inflammatory cytokines, such as TNF-α and IL-1β, contribute to blood–brain barrier disruption, neuroinflammation, and neuronal damage, leading to coma and neurological sequelae. In *Severe Malarial Anemia (SMA)*, excessive production of pro-inflammatory cytokines, coupled with dyserythropoiesis and erythrocyte destruction, contributes to the development of anemia. TNF-α, interferon-gamma (IFN-γ), and other cytokines suppress erythropoiesis and promote erythrocyte apoptosis, exacerbating anemia in malaria-infected individuals. In *Pregnancy-Associated Malaria (PAM)*, placental sequestration of infected erythrocytes triggers an inflammatory response characterized by elevated levels of pro-inflammatory cytokines and chemokines. This local inflammation contributes to placental damage, fetal growth restriction, and adverse pregnancy outcomes. Targeting dysregulated cytokine and chemokine responses with immunomodulatory agents holds promise for the treatment of severe malaria syndromes. Anti-inflammatory drugs, such as corticosteroids and TNF-α inhibitors, are being investigated for their potential to mitigate immunopathology and improve clinical outcomes. Adjunctive therapies that modulate cytokine and chemokine responses, such as intravenous immunoglobulin (IVIG) and erythropoietin, have been proposed as adjuvants to standard antimalarial treatment. These therapies aim to attenuate inflammation, support erythropoiesis, and enhance host immune responses against malaria infection^28^.

### *Anopheles* mosquito saliva biomarkers

Research on *Anopheles* mosquito saliva biomarkers is an emerging field with significant potential for enhancing malaria surveillance, diagnosis, and control efforts. *Anopheles* mosquitoes inject saliva into the host’s bloodstream during blood feeding to facilitate the acquisition of a blood meal. Mosquito saliva contains a complex mixture of proteins, enzymes, and other bioactive molecules that modulate host immune responses and facilitate successful feeding. These salivary components have emerged as potential biomarkers for monitoring mosquito biting activity and assessing human–vector contact. Salivary proteins from *Anopheles* mosquitoes can elicit immune responses in humans, leading to the production of specific antibodies against mosquito saliva. These anti-saliva antibodies serve as indicators of recent exposure to mosquito bites and can be detected using serological assays. Measuring anti-saliva antibody levels in human populations provides valuable information on mosquito biting rates, spatial and temporal patterns of vector activity, and the effectiveness of vector control interventions. Detection of anti-saliva antibodies in human blood samples can serve as a proxy measure of malaria exposure and transmission intensity. Seroprevalence studies using *Anopheles* saliva biomarkers can identify areas of high malaria transmission, prioritize resources for vector control interventions, and evaluate the impact of control measures on reducing human–vector contact. Furthermore, saliva-based assays offer a non-invasive and cost-effective approach for assessing malaria risk at the population level. *Anopheles* mosquito saliva biomarkers can also be utilized for monitoring vector populations and assessing vector behavior. By analyzing the presence of mosquito saliva in blood meals collected from human or animal hosts, researchers can estimate mosquito biting rates, host preference, and vector species composition. This information aids in targeting vector control interventions to high-risk areas and implementing tailored strategies for malaria prevention^28^.

Cross-reactivity between salivary proteins from different mosquito species may complicate the interpretation of serological data. Specificity assays and bioinformatic analyses are necessary to distinguish *Anopheles* saliva biomarkers from those of other mosquito vectors. Standardization of serological assays and validation of biomarker thresholds are essential for ensuring the accuracy and reliability of saliva-based diagnostic tests. Collaboration between researchers, public health agencies, and diagnostic companies is critical for advancing saliva biomarker research and translating findings into practical applications. The development of rapid diagnostic tests capable of detecting anti-saliva antibodies in field settings would facilitate real-time monitoring of human–vector contact and malaria transmission dynamics. Integration of saliva biomarker data into existing malaria surveillance systems would enhance the effectiveness of malaria control programs. By combining entomological data with epidemiological indicators, public health authorities can implement evidence-based interventions and track progress toward malaria elimination goals^29^.

### Revolutionizing malaria detection

Revolutionizing malaria detection has been a crucial goal in global health, given the significant impact of this disease on millions of people, especially in developing regions. Recent breakthroughs in biomarker innovation have shown promising strides toward more accurate, rapid, and accessible methods of malaria detection.

### CRISPR-based technologies

CRISPR-Cas9 is a versatile genome editing tool derived from the bacterial immune system. It consists of a guide RNA (gRNA) that directs the Cas9 nuclease to specific DNA sequences, where it induces double-strand breaks (DSBs). These breaks can be repaired by either non-homologous end joining (NHEJ) or homology-directed repair (HDR), allowing for precise gene editing or knockout. CRISPR-Cas9 has revolutionized genetic engineering, enabling targeted modifications of the genome in a wide range of organisms, including cells, plants, and animals. It has numerous applications in basic research, biotechnology, agriculture, and medicine, including gene therapy, disease modeling, and crop improvement. CRISPR interference (CRISPRi) and CRISPR activation (CRISPRa) utilize catalytically inactive Cas9 (dCas9) fused to effector domains to modulate gene expression. CRISPRi can silence gene expression by blocking transcription, while CRISPRa can activate gene expression by recruiting transcriptional activators to target genes. CRISPR-based gene regulation enables precise control over gene expression, allowing researchers to study gene function, identify therapeutic targets, and engineer cellular pathways. It has applications in disease research, synthetic biology, and biotechnology. CRISPR-based diagnostic technologies, such as SHERLOCK (Specific High-sensitivity Enzymatic Reporter UnLOCKing) and DETECTR (DNA Endonuclease Targeted CRISPR Trans Reporter), utilize Cas enzymes combined with nucleic acid detection systems to detect specific DNA or RNA sequences. Upon target recognition, the Cas enzyme cleaves reporter molecules, generating a signal that indicates the presence of the target. CRISPR-based diagnostic tools offer rapid, sensitive, and specific detection of nucleic acids, with applications in infectious disease diagnosis, pathogen detection, and POC testing. They have the potential to revolutionize healthcare by enabling rapid and accessible molecular diagnostics. CRISPR-based epigenome editing involves targeting specific epigenetic modifications, such as DNA methylation or histone modifications, to regulate gene expression without altering the underlying DNA sequence. This is achieved by fusing dCas9 with epigenetic effector domains or recruiting chromatin-modifying enzymes to target loci. CRISPR-based epigenome editing allows for precise manipulation of gene expression patterns, providing insights into epigenetic regulation and potential therapeutic interventions for diseases with epigenetic dysregulation. CRISPR-based antimicrobials utilize CRISPR-Cas systems to target and destroy specific pathogens, including bacteria and viruses. Engineered CRISPR systems can be programmed to target essential genes or virulence factors, leading to selective killing of pathogens. CRISPR-based antimicrobials hold promise for combating antimicrobial resistance and controlling infectious diseases. They offer potential alternatives to traditional antibiotics and antiviral drugs, with applications in agriculture, biodefense, and clinical therapy^30,31^.

### Nanotechnology

Nanotechnology has emerged as a promising approach for improving malaria diagnosis by offering enhanced sensitivity, specificity, and versatility in detection methods. Nanoparticles, including gold nanoparticles, quantum dots, magnetic nanoparticles, and silica nanoparticles, can be functionalized with specific ligands, antibodies, or DNA probes to target malaria biomarkers, such as *Plasmodium* nucleic acids, proteins, or metabolites. Nanoparticle-based diagnostic assays offer enhanced sensitivity and specificity for detecting malaria biomarkers in clinical samples. These assays can be adapted for various detection platforms, including colorimetric, fluorescence, electrochemical, and magnetic resonance imaging (MRI) methods. Nanopore sequencing technology enables direct, real-time sequencing of DNA or RNA molecules by passing them through a nanoscale pore. This approach can be applied to sequence *Plasmodium* genomes for genotyping and identifying drug resistance mutations. Nanopore sequencing allows for rapid and portable genotyping of malaria parasites in resource-limited settings. It provides valuable information for monitoring drug resistance, tracking transmission dynamics, and guiding treatment strategies. Nanoparticle-based biosensors integrate nanoparticles with sensing elements, such as surface plasmon resonance (SPR), field-effect transistors (FETs), or microcantilevers, to detect malaria biomarkers with high sensitivity and specificity. Nanoparticle-based biosensors offer rapid, label-free detection of malaria antigens or nucleic acids in clinical samples. These biosensors can be deployed in POC settings for on-site diagnosis and surveillance of malaria infections. Nanoparticle amplification strategies, such as nanoparticle-enhanced surface plasmon resonance (SPR) or nanoparticle-amplified immunoassays, utilize the unique optical or electrochemical properties of nanoparticles to amplify detection signals and improve assay sensitivity. Nanoparticle amplification techniques enhance the detection limits of malaria diagnostic assays, enabling the detection of low parasite densities or early-stage infections. These strategies are compatible with various detection platforms and can be integrated into existing diagnostic workflows. Nanotechnology-based sample preparation methods, such as magnetic nanoparticles for pathogen enrichment or microfluidic devices for sample processing, can improve the efficiency and sensitivity of malaria diagnostic assays. Nanotechnology-enabled sample preparation techniques streamline the workflow of malaria diagnosis, reducing turnaround times and resource requirements. These methods are particularly valuable for processing complex clinical samples, such as whole blood or saliva, in POC settings^32,33^.

### Metabolomics and proteomics

Metabolomics and proteomics are powerful omics approaches that have emerged as valuable tools for malaria diagnosis, offering insights into the host–parasite interaction and providing potential biomarkers for disease detection. Metabolomics involves the comprehensive analysis of small molecule metabolites within biological systems. In the context of malaria, metabolomic profiling can reveal changes in host and parasite metabolism in response to infection. Metabolomic profiling of plasma, serum, urine, or other biological samples can identify metabolite signatures associated with malaria infection. These biomarkers can serve as diagnostic indicators for detecting malaria. Metabolomics can elucidate metabolic pathways essential for parasite survival, providing insights into potential drug targets for antimalarial therapy. Changes in metabolite profiles before and after antimalarial treatment can be used to monitor treatment efficacy and evaluate patient response. Metabolomic analyses often employ mass spectrometry (MS) or nuclear magnetic resonance (NMR) spectroscopy to identify and quantify metabolites in biological samples. Proteomics involves the large-scale study of proteins expressed within a biological system. In malaria, proteomic analyses can characterize changes in host and parasite protein expression during infection. Proteomic profiling of serum, plasma, or other biological samples can identify proteins differentially expressed in malaria-infected individuals, serving as potential diagnostic biomarkers. Proteomics can elucidate host immune responses to malaria infection and identify parasite proteins involved in immune evasion and pathogenesis. Proteomic analyses can identify parasite proteins essential for survival, proliferation, or virulence, aiding in the discovery of novel drug targets. Proteomic approaches often involve liquid chromatography coupled with mass spectrometry (LC–MS/MS) for protein identification and quantification. Integrating metabolomic, proteomic, and genomic data can provide comprehensive insights into malaria pathogenesis and identify novel biomarkers for diagnosis and treatment. Systems biology approaches combine omics data with computational modeling to unravel complex host–parasite interactions and predict disease outcomes. Omics-based approaches enable personalized diagnosis and treatment strategies tailored to individual patient profiles, improving therapeutic efficacy and minimizing adverse effects. Standardization of sample collection and processing protocols is essential for reproducible omics analyses in malaria diagnosis. Advanced bioinformatics tools and computational algorithms are needed to analyze and interpret large-scale omics datasets effectively. Efforts are underway to develop rapid, POC diagnostic tests based on omics biomarkers for use in resource-limited settings. Rigorous validation of omics-based biomarkers is required to assess their clinical utility and reliability for malaria diagnosis^34,35^.

### AI-powered diagnostics

AI-powered diagnostics leverage AI algorithms to analyze medical data and aid in disease diagnosis. AI algorithms can analyze images of blood smears or RDTs to detect and classify malaria parasites based on their morphological features. AI algorithms can automatically detect and quantify malaria parasites in blood smears, reducing the need for manual microscopy and improving diagnostic accuracy. AI algorithms can interpret RDT results from digital images, providing rapid and objective diagnosis of malaria infections. Image-based diagnosis using AI enables rapid, scalable, and objective assessment of malaria infection, particularly in resource-limited settings where access to skilled microscopists may be limited. AI algorithms can analyze genomic data, such as NGS data or PCR results, to identify malaria species, detect drug resistance mutations, and characterize parasite populations. AI algorithms can differentiate between different *Plasmodium* species based on their genetic signatures, aiding in species-specific diagnosis and epidemiological surveillance. AI algorithms can identify genetic mutations associated with antimalarial drug resistance, helping to guide treatment decisions and monitor the emergence of drug-resistant strains. Genomic analysis using AI provides comprehensive and accurate characterization of malaria parasites, facilitating personalized treatment and informing public health interventions^36^.

AI algorithms can analyze clinical data, such as patient symptoms, laboratory test results, and demographic information, to assist in malaria diagnosis and patient management. AI algorithms can predict malaria risk and severity based on patient data, enabling early intervention and personalized treatment. AI algorithms can provide clinicians with diagnostic recommendations and treatment guidelines based on patient-specific data, improving clinical decision-making. Clinical data analysis using AI enhances diagnostic accuracy, supports evidence-based medicine, and optimizes patient outcomes by providing timely and tailored interventions. AI algorithms can integrate data from multiple sources, including imaging, genomics, clinical data, and epidemiological data, to provide a comprehensive assessment of malaria infection and transmission dynamics. AI-powered diagnostics can integrate human, animal, and environmental data to understand the complex interactions driving malaria transmission and inform integrated control strategies. AI algorithms can analyze diverse data streams to detect outbreaks, monitor disease trends, and provide early warnings of emerging threats. Integration of multiple data sources using AI enhances situational awareness, improves outbreak response, and supports evidence-based policymaking for malaria control and elimination efforts^37^.

### POC devices

Compact, portable diagnostic devices based on microfluidics and lab-on-a-chip technologies are in development. These devices enable rapid and cost-effective detection of malaria parasites, often with minimal sample volumes. They are well-suited for deployment in resource-limited settings.

### Smartphone-based diagnostics

Smartphone-based diagnostics harness the power of mobile technology to provide accessible, rapid, and user-friendly diagnostic solutions for various health conditions, including malaria. Smartphone microscopy utilizes the camera and computational capabilities of smartphones to capture and analyze images of blood smears for malaria diagnosis. With the aid of attachment accessories or specially designed microscopes, smartphones can magnify and capture high-resolution images of stained blood smears. These images can then be analyzed using image processing algorithms to detect and quantify malaria parasites. Smartphone microscopy enables decentralized malaria diagnosis, allowing healthcare workers in remote areas to perform rapid and accurate malaria tests without the need for specialized equipment or expertise. Smartphone-based RDTs involve attaching or embedding a smartphone-compatible device with the RDT strip to capture and analyze the test results. The smartphone’s camera can capture images of the RDT strip, and dedicated mobile applications can interpret the test results using image processing algorithms. These applications can provide qualitative or semi-quantitative results for malaria diagnosis. Smartphone-based RDTs offer the convenience of real-time test interpretation, reducing human error and enabling remote monitoring of malaria prevalence and treatment outcomes. Smartphone-based NATs leverage the computational power of smartphones to perform nucleic acid amplification tests (NAATs) for malaria diagnosis. By integrating miniaturized PCR or isothermal amplification technologies with smartphone platforms, users can perform molecular detection of malaria DNA or RNA. Mobile applications can analyze the fluorescence signals generated during amplification and interpret the test results. Smartphone-based NATs offer high sensitivity and specificity for malaria diagnosis, enabling detection of low-level parasitemia and species differentiation. They are particularly useful for surveillance, research, and monitoring of drug resistance^38^.

Smartphone-based diagnostic platforms can integrate with cloud-based databases, electronic health records (EHRs), and remote monitoring systems to facilitate data sharing, analysis, and decision-making. By uploading test results and patient information to centralized servers, smartphone-based diagnostics enable real-time surveillance of malaria prevalence, tracking of treatment outcomes, and rapid response to outbreaks. Connectivity features enable seamless communication between healthcare providers, researchers, and public health authorities, improving coordination and resource allocation for malaria control and elimination efforts. Smartphone-based diagnostic applications are designed with intuitive user interfaces and accessibility features to ensure ease of use for healthcare workers and patients. Mobile applications for malaria diagnosis provide step-by-step instructions, visual aids, and interactive features to guide users through the testing process. They may also offer multilingual support and compatibility with low-cost smartphone models. User-friendly interfaces enhance the adoption and usability of smartphone-based diagnostics, particularly in low-resource settings where literacy levels and technological literacy may vary^39^.

### Biosensors

Biosensors are analytical devices that combine a biological recognition element with a physicochemical transducer to detect and quantify the presence of specific biological molecules or analytes. In the context of malaria diagnosis, biosensors offer rapid, sensitive, and specific detection of malaria biomarkers, enabling POC testing and surveillance. Biosensors utilize biological molecules, such as antibodies, enzymes, nucleic acids, or whole cells, that selectively bind to target malaria biomarkers, such as *Plasmodium* antigens or nucleic acids. The biological recognition element interacts with the target analyte, generating a signal that is transduced into a measurable output, such as an electrical, optical, or thermal signal. The transducer converts the biochemical interaction between the biological recognition element and the target analyte into a quantifiable signal, which can be interpreted to determine the presence and concentration of the analyte. Optical biosensors, such as surface plasmon resonance (SPR) sensors, fluorescence-based sensors, or colorimetric assays, utilize changes in light absorption, emission, or scattering properties to detect malaria biomarkers. Electrochemical biosensors, including amperometric, potentiometric, and impedimetric sensors, measure changes in electrical properties, such as current, voltage, or impedance, resulting from the interaction between the biological recognition element and the target analyte. Mass-based biosensors, such as quartz crystal microbalance (QCM) sensors or surface acoustic wave (SAW) sensors, detect changes in mass or density caused by the binding of the target analyte to the sensor surface. Biosensors can detect malaria antigens, such as HRP2, pLDH, or aldolase, in patient blood samples. These biosensors offer rapid and sensitive diagnosis of malaria, particularly in settings where microscopy may be unavailable or impractical. Biosensors can detect malaria DNA or RNA using nucleic acid amplification techniques, such as PCR or isothermal amplification, combined with electrochemical or optical detection methods. These biosensors enable species-specific diagnosis and detection of drug resistance mutations^11^.

Biosensors offer rapid detection of malaria biomarkers, providing actionable results within minutes to hours, depending on the assay format and detection method. Biosensors can achieve high sensitivity and specificity for malaria diagnosis, enabling accurate detection of low-level parasitemia and differentiation between *Plasmodium* species. Miniaturized and portable biosensor devices enable decentralized testing at the point of care, reducing turnaround times and improving access to timely diagnosis. Biosensors can be multiplexed to detect multiple malaria biomarkers simultaneously, enhancing diagnostic accuracy and providing comprehensive information about infection status and drug resistance. Robust validation and standardization of biosensor assays are essential to ensure accuracy, reliability, and reproducibility across different settings and populations. Despite advances in biosensor technology, cost-effective manufacturing and distribution models are needed to make biosensors accessible and affordable in resource-limited settings. Integration of biosensors into existing healthcare systems requires infrastructure support, training of healthcare workers, and regulatory approval to ensure effective implementation and sustainable use^56^.

### Biomarker innovation

In the context of malaria, biomarker innovation is crucial for the development of accurate, rapid, and cost-effective diagnostic tools, as well as for monitoring treatment efficacy and disease progression. Malaria biomarkers can be derived from various sources, including the host’s immune response and the parasite’s genetic and metabolic activities. Here are some key areas of malaria biomarker innovation:

### Genetic biomarkers

Genetic biomarkers are specific genetic variations or signatures that can be used to indicate the presence of a disease, predict disease progression, or guide treatment decisions. In the context of malaria, genetic biomarkers play a crucial role in understanding parasite biology, monitoring drug resistance, and informing malaria control strategies. Genetic biomarkers specific to different *Plasmodium* species, such as *Plasmodium falciparum*, *Plasmodium vivax*, *Plasmodium malariae*, and *Plasmodium ovale*, can be used to distinguish between different types of malaria infections. Species-specific genes, such as the HRP2 gene for *P. falciparum* or the Duffy antigen receptor for chemokines (DARC) gene for *P. vivax*, are commonly used as biomarkers for species identification in diagnostic assays and epidemiological studies. Genetic mutations associated with antimalarial drug resistance serve as biomarkers for monitoring the emergence and spread of drug-resistant strains. Drug resistance markers, such as mutations in the *P. falciparum* chloroquine resistance transporter (pfcrt) gene or the *P. falciparum* multidrug resistance 1 (pfmdr1) gene, are used to track resistance to antimalarial drugs and guide treatment strategies^40^.

Genetic mutations in the kelch13 (K13) propeller domain of *P. falciparum* have been associated with artemisinin resistance, posing a significant threat to malaria control efforts. K13 mutations serve as biomarkers for monitoring artemisinin resistance and identifying regions where resistance is emerging, enabling targeted interventions to prevent the spread of resistant parasites. Genetic biomarkers can be used in population genetics studies to analyze the genetic diversity and structure of malaria parasite populations. Population genetic analyses provide insights into the transmission dynamics, evolutionary history, and migration patterns of malaria parasites, informing the design and implementation of malaria control programs. Genetic variations in host genes involved in immune responses, such as human leukocyte antigen (HLA) genes or cytokine genes, can influence susceptibility to malaria infection and disease severity. Host genetic biomarkers contribute to our understanding of host–parasite interactions and can help identify individuals at higher risk of severe malaria, informing targeted interventions and personalized treatment approaches. High-throughput genomic techniques, such as next-generation sequencing (NGS) or genome-wide association studies (GWAS), enable comprehensive analysis of genetic variation across the malaria parasite genome or the human genome. These techniques facilitate the discovery of novel genetic biomarkers associated with malaria infection, drug resistance, or disease outcomes, providing valuable insights into malaria pathogenesis and informing the development of new interventions^41^.

### Protein biomarkers

Protein biomarkers are specific proteins or peptides that can be measured in biological samples to indicate the presence of a disease, monitor disease progression, or predict treatment response. In the context of malaria, protein biomarkers play a crucial role in understanding host–parasite interactions, diagnosing infection, and assessing disease severity. Proteins produced by the malaria parasite, such as (HRP2, pLDH, and aldolase, serve as biomarkers for malaria infection. These parasite-derived proteins are commonly used in RDTs to detect malaria antigens in patient blood samples, providing rapid and sensitive diagnosis of malaria. Inflammatory biomarkers, such as cytokines, chemokines, and acute phase proteins, are produced by the host immune response to malaria infection. Measurement of inflammatory biomarkers, such as TNF-α, IL-6, and CRP, can provide insights into disease pathogenesis, assess disease severity, and monitor treatment response in malaria patients^42^.

Endothelial activation markers, such as soluble intercellular adhesion molecule-1 (sICAM-1) and soluble vascular cell adhesion molecule-1 (sVCAM-1), are proteins released by endothelial cells in response to inflammation and vascular injury. Elevated levels of endothelial activation markers in malaria patients are associated with endothelial dysfunction, microvascular sequestration of infected erythrocytes, and severe malaria syndromes, such as cerebral malaria and severe malarial anemia. Biomarkers of organ dysfunction, such as serum creatinine, blood urea nitrogen (BUN), and lactate, reflect impaired organ function and metabolic derangements in severe malaria. Measurement of biomarkers of organ dysfunction helps assess the severity of malaria complications, such as renal failure, metabolic acidosis, and cerebral malaria, guiding clinical management and supportive care interventions. Hemolytic biomarkers, such as plasma hemoglobin, haptoglobin, and bilirubin, are indicators of hemolysis and red blood cell destruction in malaria-infected individuals. Elevated levels of hemolytic biomarkers are associated with severe malaria complications, including hemoglobinuria, jaundice, and organ damage, and can help identify patients at risk of severe disease and adverse outcomes. Proteomic profiling techniques, such as mass spectrometry (MS) and protein microarrays, enable comprehensive analysis of protein expression and modification patterns in malaria-infected individuals. Proteomic profiling of plasma, serum, or other biological samples from malaria patients can identify novel protein biomarkers associated with disease severity, treatment response, and immune status, providing insights into malaria pathogenesis and informing biomarker discovery efforts^43^.

### Metabolic biomarkers

Metabolic biomarkers are small molecules or metabolites that reflect the biochemical changes occurring in an organism’s metabolism in response to disease, environmental factors, or therapeutic interventions. In the context of malaria, metabolic biomarkers offer insights into parasite biology, host responses, and disease progression. Hemoglobin metabolism is altered during malaria infection, leading to the release of hemoglobin breakdown products, such as heme and free iron, into the bloodstream. Measurement of hemoglobin metabolites, such as plasma heme and serum iron, provides insights into hemolysis, erythrocyte destruction, and iron homeostasis in malaria-infected individuals. Malaria infection induces oxidative stress in host cells, resulting in the generation of reactive oxygen species (ROS) and oxidative damage to lipids, proteins, and DNA. Biomarkers of oxidative stress, such as malondialdehyde (MDA), glutathione (GSH), and superoxide dismutase (SOD), serve as indicators of redox imbalance and cellular damage in malaria patients. Malaria parasites rely on host glucose and energy metabolism to fuel their growth and replication within infected erythrocytes. Metabolites involved in energy metabolism, such as glucose, lactate, and adenosine triphosphate (ATP), can serve as biomarkers of parasite metabolism, disease severity, and treatment response in malaria-infected individuals^44^.

Malaria parasites undergo hepatic stages during their life cycle, leading to liver inflammation and dysfunction in some cases. Biomarkers of liver dysfunction, such as alanine aminotransferase (ALT), aspartate aminotransferase (AST), and bilirubin, can indicate hepatocyte injury and liver dysfunction in severe malaria cases. Metabolomic profiling techniques, such as nuclear magnetic resonance (NMR) spectroscopy and mass spectrometry (MS), enable comprehensive analysis of metabolite profiles in biological samples from malaria-infected individuals. Metabolic profiling of plasma, serum, urine, or other biological fluids can identify novel metabolic biomarkers associated with malaria infection, severity, and treatment outcomes, providing insights into malaria pathogenesis and host–parasite interactions. Severe malaria can lead to multi-organ dysfunction, including renal failure, metabolic acidosis, and cerebral malaria. Metabolic biomarkers associated with organ dysfunction, such as serum creatinine, BUN, lactate, and ketone bodies, can help assess disease severity, guide clinical management, and monitor treatment response in malaria patients^45^.

### Host immune response

The host immune response to malaria infection is a complex interplay between the parasite, the host immune system, and environmental factors. Understanding the dynamics of the immune response is crucial for elucidating malaria pathogenesis, developing effective vaccines, and improving treatment strategies. Upon infection with malaria parasites, innate immune cells, such as macrophages, dendritic cells, and natural killer (NK) cells, recognize pathogen-associated molecular patterns (PAMPs) and initiate an inflammatory response. Innate immune cells release pro-inflammatory cytokines, such as TNF-α, interleukin-1 (IL-1), and IFN-γ, to activate immune effector mechanisms and recruit other immune cells to the site of infection. Macrophages and other phagocytic cells engulf and eliminate malaria-infected erythrocytes, contributing to parasite clearance and resolution of the acute infection. Dendritic cells present malaria antigens to T cells in the draining lymph nodes, initiating an adaptive immune response. CD4+ T cells differentiate into effector T helper (Th) cell subsets, such as Th1, Th2, and Th17 cells, in response to specific malaria antigens. CD8+ cytotoxic T cells (CTLs) are activated to kill infected cells. B cells produce antibodies (immunoglobulins) targeting malaria antigens, including surface antigens on infected erythrocytes, merozoites, and sporozoites. Antibodies play a crucial role in controlling parasitemia and preventing reinfection^46^.

Excessive or dysregulated immune activation can contribute to immunopathogenesis and tissue damage in severe malaria syndromes, such as cerebral malaria, severe malarial anemia, and acute respiratory distress syndrome (ARDS). Pro-inflammatory cytokines and activated immune cells induce endothelial activation, adhesion molecule expression, and microvascular sequestration of infected erythrocytes, contributing to tissue hypoxia and organ dysfunction. Malaria parasites employ various immune evasion mechanisms to evade host immune responses, including antigenic variation, sequestration in deep tissues, and modulation of host immune signaling pathways. Successful resolution of malaria infection leads to the development of long-lasting immune memory against specific parasite antigens. Vaccines targeting key malaria antigens, such as circumsporozoite protein (CSP), merozoite surface proteins (MSPs), and apical membrane antigen 1 (AMA1), aim to induce protective immune responses and prevent infection or severe disease. Therapeutic interventions targeting the immune response, such as anti-inflammatory drugs or immunomodulatory agents, have been investigated as adjunctive treatments for severe malaria to modulate excessive immune activation and mitigate immunopathology. Immunotherapies, including monoclonal antibodies targeting specific cytokines or immune checkpoints, are being explored as potential treatments to enhance host immune responses and improve outcomes in severe malaria^47^.

### MicroRNA signatures

MicroRNAs (miRNAs) are small non-coding RNA molecules that play crucial roles in post-transcriptional gene regulation by binding to target messenger RNA (mRNA) molecules, leading to mRNA degradation or translational repression. In the context of malaria, miRNAs have emerged as important regulators of host–parasite interactions, immune responses, and disease pathogenesis. Differential expression of host miRNAs in response to malaria infection regulates immune cell activation, cytokine production, and inflammatory responses. Malaria parasites release miRNA-containing exosomes or small RNA molecules that modulate host immune responses and promote parasite survival. Circulating miRNAs in plasma or serum have been identified as potential biomarkers for malaria diagnosis, disease severity, and treatment response. Tissue-specific miRNA signatures in peripheral blood cells, liver, spleen, or brain tissues may reflect malaria-induced organ damage and dysfunction. Differential expression of specific miRNAs correlates with disease severity, clinical outcomes, and risk of complications in malaria patients. miRNA profiles may predict treatment outcomes, therapeutic responses, and susceptibility to drug resistance in malaria-infected individuals. Malaria parasites modulate host immune responses and evade immune detection by releasing miRNAs that suppress host immune effectors, inhibit cytokine signaling pathways, or interfere with antigen presentation. Host miRNAs targeted by malaria parasites contribute to immune evasion, parasite persistence, and chronic infection. Host genetic polymorphisms in miRNA genes or miRNA target sites may influence susceptibility to malaria infection, disease progression, and treatment outcomes. miRNA-mediated epigenetic modifications, such as DNA methylation or histone modifications, regulate gene expression patterns associated with malaria susceptibility or resistance. Manipulation of miRNA expression levels or activity using synthetic miRNA mimics, antagomiRs, or miRNA inhibitors represents a potential therapeutic approach for modulating immune responses and controlling parasite replication in malaria. Identification of miRNA targets and pathways involved in malaria pathogenesis may lead to the development of novel antimalarial drugs targeting host–parasite interactions^48,49^.

### Biosensors and POC devices

Biosensors and POC devices play critical roles in the rapid and accurate diagnosis of malaria, particularly in resource-limited settings where access to traditional laboratory facilities may be limited. Biosensors are analytical devices that integrate a biological sensing element (e.g. antibodies, enzymes, or nucleic acids) with a physicochemical transducer to detect specific biomolecules (e.g. malaria antigens or nucleic acids) in patient samples. Biosensors for malaria diagnosis include optical biosensors (e.g. surface plasmon resonance), electrochemical biosensors (e.g. amperometric or impedimetric sensors), and mass-based biosensors (e.g. quartz crystal microbalance). Biosensors offer rapid, sensitive, and specific detection of malaria biomarkers, enabling timely diagnosis and treatment initiation. They can be miniaturized, portable, and suitable for POC testing in remote or resource-limited settings. POC devices are portable diagnostic tools designed to deliver rapid and user-friendly testing at or near the patient’s location, without the need for centralized laboratory facilities or specialized training. POC devices for malaria diagnosis include rapid diagnostic tests (RDTs), molecular diagnostic platforms (e.g. loop-mediated isothermal amplification, PCR miniaturized systems), smartphone-based diagnostics, and handheld microscopy devices. POC devices enable decentralized testing, rapid results delivery, and immediate treatment initiation, improving patient outcomes and reducing the burden on healthcare infrastructure. They are particularly valuable in remote or underserved areas where laboratory access is limited. Some POC devices utilize biosensor technology for detection and quantification of malaria biomarkers. For example, RDTs incorporate lateral flow immunoassays as biosensing elements to detect malaria antigens. Integration of biosensors with POC devices enhances diagnostic accuracy, sensitivity, and specificity, leading to improved performance compared to traditional methods. Biosensor-based POC devices often feature user-friendly interfaces, visual readouts, or digital displays to facilitate interpretation of test results by healthcare providers or end-users. Robust validation and standardization of biosensor-based POC devices are essential to ensure accuracy, reliability, and consistency of test results across different settings and populations. Despite their numerous advantages, biosensor-based POC devices may face challenges related to cost, accessibility, and sustainability in resource-limited settings. Regulatory approval and quality assurance processes are necessary to ensure compliance with safety and performance standards for biosensor-based POC devices^50,51^.

### Next-generation sequencing (NGS)

Next-generation sequencing (NGS) revolutionized the field of genomics by enabling high-throughput, massively parallel DNA sequencing, leading to rapid advancements in genomic research, diagnostics, and personalized medicine. NGS platforms simultaneously sequence millions of DNA fragments in parallel, generating vast amounts of sequencing data in a single run. NGS techniques, such as Illumina sequencing, employ sequencing-by-synthesis chemistry to determine the nucleotide sequence of DNA fragments by detecting fluorescently labeled nucleotides as they are incorporated into a growing DNA strand. NGS enables whole-genome sequencing of malaria parasites, providing insights into genetic diversity, population structure, and evolution of drug resistance. NGS facilitates surveillance of drug-resistant malaria strains by identifying single nucleotide polymorphisms (SNPs) and genetic markers associated with resistance to antimalarial drugs. NGS-based approaches, such as genome-wide association studies (GWAS) and population genomics analyses, help elucidate the genetic basis of malaria susceptibility, host–parasite interactions, and adaptation to diverse environments. RNA sequencing (RNA-seq) using NGS enables transcriptomic profiling of malaria parasites and host immune responses, elucidating gene expression patterns, regulatory networks, and pathways involved in disease pathogenesis. NGS-based techniques, such as chromatin immunoprecipitation sequencing (ChIP-seq) and bisulfite sequencing, allow characterization of epigenetic modifications, chromatin accessibility, and DNA methylation patterns in malaria parasites and host cells^52^.

NGS-based assays offer sensitive and specific detection of malaria parasites, species identification, and monitoring of drug resistance markers in clinical samples, enhancing diagnostic accuracy and surveillance efforts. NGS enables genomic epidemiological studies to track the spread of malaria strains, identify transmission hotspots, and investigate outbreaks, informing targeted interventions and control strategies. NGS facilitates the identification of host genetic factors associated with malaria susceptibility, severity, and treatment outcomes, paving the way for personalized treatment approaches and precision medicine strategies. Advances in single-cell sequencing technologies enable the analysis of individual malaria parasites or host immune cells, providing insights into cellular heterogeneity, clonal dynamics, and host–pathogen interactions. Long-read sequencing platforms, such as Pacific Biosciences (PacBio) and Oxford Nanopore Technologies (ONT), offer advantages for sequencing complex regions of the malaria genome, resolving structural variants, and phasing haplotypes. NGS-based metagenomic approaches allow the characterization of microbial communities, including the human microbiome and mosquito gut microbiota, shedding light on their role in malaria transmission and pathogenesis^53^.

### Imaging biomarkers

Imaging biomarkers are quantitative measurements derived from medical imaging techniques that provide information about normal biological processes, pathophysiological changes, or response to therapy. In the context of malaria, imaging biomarkers play a crucial role in understanding disease pathogenesis, monitoring treatment efficacy, and guiding clinical management. Light microscopy of blood smears is the gold standard for malaria diagnosis, allowing visualization of malaria parasites within red blood cells (RBCs) and determination of parasite species and parasitemia. Molecular imaging techniques, such as positron emission tomography (PET) or magnetic resonance imaging (MRI) with molecular probes, enable visualization of specific molecular targets or metabolic processes associated with malaria infection. Quantification of parasite density from blood smears or imaging studies provides a measure of disease severity and treatment response in malaria-infected individuals. Hemozoin, a byproduct of hemoglobin digestion by malaria parasites, can be visualized using imaging techniques, serving as a biomarker of parasite burden and disease activity. Imaging studies, such as ultrasound, computed tomography (CT), or MRI, reveal pathological changes in organs affected by severe malaria complications, including the brain (cerebral malaria), lungs (acute respiratory distress syndrome), liver, spleen, and kidneys. Vascular sequestration of malaria-infected RBCs in deep tissues and microvasculature can be visualized using imaging techniques, providing insights into disease pathogenesis and organ dysfunction^54^.

Serial imaging studies allow monitoring of parasite clearance and resolution of parasitemia in response to antimalarial treatment, guiding clinical management and therapeutic decisions. Imaging biomarkers assess tissue recovery and resolution of organ dysfunction following successful treatment of severe malaria complications, aiding in prognostication and patient outcomes. Imaging biomarkers are used in preclinical malaria models, such as rodent models or non-human primates, to study disease pathogenesis, evaluate novel therapeutics, and assess vaccine efficacy. Advanced imaging techniques, combined with omics approaches, facilitate the discovery of novel imaging biomarkers associated with malaria infection, disease progression, and treatment response. Access to advanced imaging technologies may be limited in resource-limited settings where malaria is endemic, posing challenges for diagnosis and clinical management. Standardized protocols and interpretation criteria are needed for consistent and reproducible imaging biomarker assessment across different imaging modalities and clinical settings. Integration of imaging biomarkers into routine clinical care requires multidisciplinary collaboration, infrastructure support, and training of healthcare providers to ensure appropriate utilization and interpretation of imaging studies^55^.

### ML and AI for diagnosis

ML and AI techniques have shown promising applications in malaria diagnosis, offering automated, accurate, and scalable solutions for detecting malaria parasites, predicting disease outcomes, and guiding clinical decision-making. ML algorithms can analyze digitized blood smear images to automatically detect and quantify malaria parasites, species classification, and parasitemia estimation, reducing the need for manual examination by trained microscopists. AI algorithms can interpret *Rapid Diagnostic Tests (RDT)* results from digital images captured using smartphones or portable imaging devices, providing rapid and objective diagnosis of malaria infection in remote or resource-limited settings. ML models extract relevant features from microscopy images, such as parasite morphology, texture, or spatial distribution, to differentiate between infected and uninfected RBCs and classify malaria species. Supervised learning algorithms, such as support vector machines (SVM), random forests, or convolutional neural networks (CNNs), are trained on labeled datasets to classify malaria-infected samples and distinguish between different parasite species. ML models integrate clinical data, such as patient demographics, symptoms, and laboratory test results, with imaging data to improve diagnostic accuracy and predict disease severity or treatment response. AI techniques combine imaging data with genomic, transcriptomic, or proteomic data to identify biomarkers associated with malaria infection, drug resistance, or host immune responses, enhancing our understanding of disease pathogenesis and treatment outcomes^11^.

ML-based algorithms deployed on portable devices or cloud-based platforms enable real-time analysis of diagnostic tests, providing rapid results and decision support for healthcare providers at the point of care. AI-driven decision support systems integrate patient data, imaging results, and clinical guidelines to assist healthcare providers in malaria diagnosis, treatment selection, and patient management. ML algorithms analyze satellite imagery, climate data, and geographic information systems (GIS) to predict malaria transmission dynamics, identify high-risk areas, and guide targeted interventions for vector control and disease surveillance. AI models use time-series data and ML algorithms to forecast malaria incidence, outbreak detection, and response planning, supporting public health efforts to control and eliminate malaria. ML models require high-quality, well-annotated datasets for training and validation, which may be limited in resource-limited settings or biased toward certain populations. Black-box nature of some ML algorithms may hinder interpretability and trust among healthcare providers, necessitating transparent model architectures and explainable AI approaches. ML-based diagnostic tools need regulatory approval, clinical validation, and integration into healthcare systems to ensure safety, efficacy, and widespread adoption in clinical practice^56^.

### Volatile organic compounds (VOCs)

Volatile organic compounds (VOCs) are organic chemicals that have high vapor pressure and low boiling points, enabling them to easily evaporate into the air at room temperature. VOCs originate from various sources, including natural processes, human activities, and industrial emissions. In the context of malaria, VOCs have been investigated as potential biomarkers for malaria diagnosis, vector control, and disease surveillance. Malaria parasites metabolize host hemoglobin and produce VOCs as metabolic byproducts, which can be detected in exhaled breath or skin emanations of infected individuals. VOC profiles may reflect host immune responses to malaria infection, including oxidative stress, inflammation, and tissue damage, providing insights into disease pathogenesis and severity. VOCs emitted from human skin and breath contribute to human odor, which can attract malaria vectors, such as *Anopheles* mosquitoes, facilitating host-seeking behavior and blood feeding. Certain VOCs, such as DEET (*N*,*N*-diethyl-meta-toluamide) or picaridin, are commonly used as mosquito repellents to deter mosquito biting and reduce malaria transmission risk. VOCs in exhaled breath samples have been investigated as potential non-invasive biomarkers for malaria diagnosis, offering a rapid and sensitive diagnostic approach for screening and surveillance. VOCs released from human skin, sweat, or urine samples may contain specific volatile metabolites associated with malaria infection, which can be analyzed for diagnostic purposes^57^.

VOC-based traps or attractants are used in mosquito surveillance programs to monitor vector populations, assess vector density, and evaluate the effectiveness of vector control interventions. VOC signatures in environmental air samples or soil emissions may indicate the presence of malaria transmission hotspots, guiding targeted control efforts and public health interventions. *Gas Chromatography–Mass Spectrometry (GC–MS)* techniques enable identification and quantification of VOCs in complex biological or environmental samples, providing detailed VOC profiles for malaria research and surveillance. Electronic nose devices, equipped with arrays of chemical sensors, can detect and discriminate VOC patterns associated with malaria infection, offering potential POC diagnostic tools for resource-limited settings.

Standardized protocols and reference databases are needed for VOC sampling, analysis, and interpretation to ensure consistency and reproducibility of results. VOC profiles may be influenced by factors such as diet, medication, or co-infections, leading to variability and potential cross-reactivity in diagnostic assays. Ethical issues related to privacy, consent, and data sharing must be addressed in VOC-based diagnostic studies involving human subjects, particularly in vulnerable populations^58^.

### Comprehensive analysis of the current state of molecular diagnostics in malaria

Malaria, caused by *Plasmodium* parasites transmitted through *Anopheles* mosquitoes, remains a significant global health challenge, particularly in tropical and subtropical regions. Molecular diagnostics have revolutionized the field of malaria detection, offering improved sensitivity, specificity, and the ability to detect low parasite densities, which is crucial for effective disease management and control. This analysis explores the current state of molecular diagnostics in malaria, highlighting key technologies, challenges, and future prospects. Widely used for its high sensitivity and specificity, Real-time PCR (qPCR) detects and quantifies *Plasmodium* DNA in clinical samples. It allows for rapid identification of species and assessment of parasite load, aiding in treatment decisions and surveillance efforts. Nested PCR offers increased sensitivity by amplifying specific DNA sequences in two successive PCR reactions, enabling detection even at low parasite densities. Loop-mediated isothermal amplification (LAMP) is gaining popularity due to its simplicity, speed, and tolerance to inhibitors present in clinical samples. It amplifies DNA under isothermal conditions, making it suitable for resource-limited settings where sophisticated laboratory infrastructure is lacking. Microarrays enable simultaneous detection of multiple *Plasmodium* species and genetic markers associated with drug resistance. They provide a comprehensive profile of parasite diversity and evolution. Next-generation sequencing (NGS): Facilitates whole-genome sequencing of *Plasmodium* parasites, aiding in understanding transmission dynamics, genetic diversity, and emergence of drug resistance. It promises personalized medicine approaches and informs public health strategies. Identification of specific proteins and metabolites associated with malaria infection provides insights into disease pathogenesis and potential diagnostic targets. Utilize biomarker detection for rapid, POC testing, enhancing accessibility and timely diagnosis in remote areas^57,58^.

### Challenges and future prospects of biomarkers for malaria

Biomarkers have emerged as invaluable tools in the battle against malaria, offering insights into diagnosis, severity assessment, and treatment efficacy^59^. However, while biomarkers have greatly improved our understanding of the disease, several challenges and promising prospects lie ahead. Biomarker standardization across different laboratories and settings remains a significant challenge. Variability in sample collection, processing, and analysis can affect the reliability and comparability of results. Asymptomatic and submicroscopic malaria infections pose a significant challenge for biomarker-based diagnosis and surveillance. Identifying individuals with low parasite densities or those who are carriers but show no symptoms is critical for disease control^60^. As malaria parasites develop resistance to commonly used antimalarial drugs, biomarkers for drug resistance are crucial. Identifying genetic markers associated with resistance and developing rapid tests for these markers are essential for effective treatment^61^. Biomarker-based diagnostic techniques often require sophisticated equipment and trained personnel, making them less accessible in resource-limited settings where malaria is most prevalent^62^.

NGS technologies hold immense promise for the discovery of novel biomarkers. They enable the comprehensive analysis of parasite genomes and transcriptomes, facilitating the identification of genetic markers associated with drug resistance and virulence^63^. Advances in nanotechnology have the potential to revolutionize malaria diagnostics. Nanoscale devices and biosensors can provide rapid and sensitive detection of malaria biomarkers at low cost, making them suitable for use in resource-limited regions^64^. The concept of personalized medicine in malaria is gaining traction. Biomarkers can aid in tailoring treatment regimens to the individual patient, considering factors like genetic susceptibility, drug metabolism, and immune response^65^. Biomarkers are instrumental in vaccine development, aiding in the assessment of vaccine efficacy, identification of immune correlates of protection, and prediction of vaccine success in different populations^66^. Combining genomics, proteomics, and metabolomics offers a holistic view of the malaria infection and the host response. Integrated multi-omics approaches can uncover novel biomarkers and therapeutic targets^67^. POC diagnostic devices for malaria are continually improving. These user-friendly, rapid tests are vital for early diagnosis and can be used in remote areas with limited healthcare infrastructure^55^. The application of AI and ML to analyze large datasets can help identify patterns and correlations among biomarkers, leading to the discovery of more accurate and predictive indicators of infection and disease severity^68^.

### Recommendations

Keep abreast of the latest developments in malaria detection technologies and biomarker innovation by regularly checking scientific journals, reputable health organizations, and research institutions. This will help you stay informed about breakthroughs and emerging trends in the field. Establish collaborations with research institutions, universities, or organizations that are actively involved in malaria research. Collaborative efforts can provide access to cutting-edge research, expertise, and potential opportunities for partnerships in biomarker innovation. Investigate funding opportunities provided by government agencies, non-profit organizations, and foundations that support malaria research and biomarker innovation. Securing funding can facilitate the development and implementation of innovative projects in this field.

Attend conferences, seminars, and workshops related to malaria, infectious diseases, and biomarker research. Engaging with experts in the field can offer valuable insights, networking opportunities, and potential collaborations. Explore the feasibility of implementing POC diagnostic technologies for malaria in regions where access to traditional laboratory facilities is limited. Investigate partnerships with companies or organizations that specialize in developing rapid and portable diagnostic devices. Stay open to adopting emerging technologies such as CRISPR-based diagnostics, AI, and innovative imaging techniques. These technologies have the potential to revolutionize malaria detection and monitoring. Encourage multidisciplinary collaborations that involve experts from various fields, including molecular biology, engineering, data science, and public health. Cross-disciplinary collaboration can lead to holistic and innovative solutions.

Advocate for public awareness and education campaigns to enhance understanding of malaria, its symptoms, and the importance of early detection. Public engagement can contribute to increased support for research and development efforts. Align your efforts with global health initiatives and partnerships that aim to combat malaria. Supporting and participating in international collaborations can amplify the impact of biomarker innovations on a broader scale. Invest in training programs and capacity building initiatives, especially in regions heavily affected by malaria. Building local expertise in biomarker research and detection technologies can contribute to sustainable solutions.

Table 1 shows Summary of Current Malaria Diagnostic Methods, Table 2 shows Commonly Used Malaria Biomarkers and their Applications, Table 3 shows Emerging Technologies for Malaria Diagnosis and Table 4 shows Challenges and Opportunities in Biomarker Innovation for Malaria Detection (provided by the authors).

**Table 1:** Summary of current malaria diagnostic methods.

Diagnostic Method	Principle	Sensitivity	Specificity	Advantages	Limitations
Microscopy	Visual detection of parasites	High	Variable	Low cost, widely available, species identification	Operator-dependent, time-consuming
Rapid Diagnostic Tests	Immunochromatographic assays	Moderate to high	Moderate to high	Rapid results, minimal training required	Limited sensitivity in low parasitemia, antigen persistence
Molecular Assays	Polymerase chain reaction (PCR)	High	High	Highly sensitive and specific, quantification	Equipment and expertise required, cost

**Table 2:** Commonly used malaria biomarkers and their applications.

Biomarker	Source	Detection Method	Applications
Histidine-Rich Protein 2 (HRP2)	Malaria parasites	RDTs, ELISA, Lateral flow assays	Diagnosis, monitoring treatment response
Lactate Dehydrogenase (LDH)	Malaria parasites	RDTs, ELISA, Colorimetric assays	Diagnosis, species differentiation, assessing severity
Aldolase	Malaria parasites	RDTs, ELISA, Colorimetric assays	Diagnosis, monitoring treatment response
Plasmodium DNA/RNA	Malaria parasites	PCR, NGS, LAMP	Diagnosis, genotyping, detecting drug resistance markers

**Table 3:** Emerging technologies for malaria diagnosis.

Technology	Principle	Advantages	Limitations
CRISPR-Based Diagnostics	CRISPR-Cas nucleic acid detection	High sensitivity, specificity, rapid results	Equipment and expertise required, cost
Nanotechnology	Nanoparticle-based assays, sensors	Miniaturization, portability, high-throughput	Standardization, scalability, cost
Metabolomics	Profiling metabolic signatures	Non-invasive, high-throughput, biomarker discovery	Data analysis, sample collection, validation
Artificial Intelligence	Machine learning, deep learning	Pattern recognition, predictive modeling	Data quality, interpretability, algorithm bias

**Table 4:** Challenges and opportunities in biomarker innovation for malaria detection.

Challenges	Opportunities
Limited sensitivity of current tests in low parasitemia settings	Development of ultrasensitive diagnostic assays
Cost and infrastructure constraints in resource-limited settings	Innovation in low-cost, point-of-care technologies
Antigen persistence leading to false-positive results	Identification of novel biomarkers for early detection
Lack of standardization and quality control measures	Collaborative efforts to establish guidelines and reference materials

## Conclusion

The quest for reliable biomarkers in the realm of malaria infection has witnessed significant strides, showcasing a diverse array of markers associated with both the parasite and the host immune response. These biomarkers have demonstrated immense potential in aiding early diagnosis, understanding disease pathogenesis, monitoring treatment efficacy, and informing public health interventions aimed at malaria control and eradication. Established biomarkers like parasite density, antigen-based tests, and molecular assays such as PCR have laid a solid foundation for malaria diagnosis. However, the emergence of host-derived biomarkers, including cytokines, chemokines, microRNAs, and metabolites, has added depth to our understanding of the complex interplay between the parasite and the host immune system. These host biomarkers offer insights into the immunological responses and underlying mechanisms of disease progression, presenting new avenues for diagnostic and therapeutic exploration.

Nevertheless, the journey toward effectively utilizing these biomarkers faces challenges. Variability in biomarker expression among different malaria species, clinical stages, and diverse host populations necessitates meticulous validation, standardization, and consideration of confounding factors. Additionally, the translation of promising biomarkers into practical clinical applications, particularly in resource-limited settings, demands innovative approaches and robust, user-friendly diagnostic tools. The integration of cutting-edge technologies like high-throughput omics, bioinformatics, and AI holds immense promise in accelerating biomarker discovery and enhancing the accuracy and accessibility of diagnostics. These advancements not only facilitate the identification of novel biomarkers but also pave the way for the development of rapid, POC tests crucial for timely and accurate malaria diagnosis. Furthermore, the multi-faceted utility of biomarkers extends beyond diagnostics, encompassing their potential in guiding vaccine development, assessing drug efficacy, and informing public health strategies for malaria control and elimination. The synergy between biomarker research and these broader applications signifies a holistic approach toward combatting malaria.

The relationship between existing techniques for using biomarkers in malaria diagnosis and the possibilities for the future represents a dynamic interplay between established methodologies and the potential for transformative advancements. Current diagnostic methods, such as rapid tests, microscopy, and molecular techniques, have significantly improved malaria detection and patient care, especially in resource-limited settings. However, these methods come with their own set of challenges, including sensitivity issues, the need for skilled personnel, and limited applicability in certain contexts. The future of malaria diagnosis hinges on a multi-faceted approach that combines technological innovation, data-driven insights, and collaborative efforts. As researchers and healthcare professionals continue to push the boundaries of biomarker discovery and diagnostic technologies, the goal is to create a more resilient and adaptable toolkit for combating malaria, ultimately leading to improved patient outcomes and enhanced global health security. The ongoing evolution of diagnostic techniques for malaria underscores the importance of staying at the forefront of scientific and technological advancements to address the complex challenges posed by this infectious disease.

## Ethical approval

Not applicable.

## Consent

Not applicable.

## Source of funding

This paper received no specific grant from any funding agency in the public, commercial, or not-for-profit sectors.

## Author contribution

Emmanuel performed the following roles: conceptualization, methodology, supervision, draft writing, editing and approval before submission.

## Conflicts of interest disclosure

The authors declare no conflicts of interest.

## Research registration unique identifying number (UIN)

Not applicable.

## Guarantor

The guarantor is Emmanuel Ifeanyi Obeagu; e-mail: emmanuelobeagu@yahoo.com; ORCID: 0000-0002-4538-0161.

## Data availability statement

Not applicable.

## Provenance and peer review

Not applicable.
